# Azacitidine Omega-3 Self-Assemblies: Synthesis, Characterization, and Potent Applications for Myelodysplastic Syndromes

**DOI:** 10.3390/ph14121317

**Published:** 2021-12-17

**Authors:** Milad Baroud, Elise Lepeltier, Yolla El-Makhour, Nolwenn Lautram, Jerome Bejaud, Sylvain Thepot, Olivier Duval

**Affiliations:** 1 Micro & Nanomedecines Translationnelles (MINT), Inserm, The National Center for Scientific Research (CNRS), SFR ICAT, University of Angers, 49000 Angers, France; milad.baroud@univ-angers.fr (M.B.); elise.lepeltier@univ-angers.fr (E.L.); nolwenn.lautram@univ-angers.fr (N.L.); jerome.bejaud@univ-angers.fr (J.B.); 2 Environmental Health Research Lab, Faculty of Science, Lebanese University, Nabatieh 1700, Lebanon; yollamakhour@yahoo.com; 3 Department of Hematology, University Hospital of Angers, 49933 Angers, France; Sylvain.Thepot@chu-angers.fr; 4 Federation Hospital of Universitaire Grand Ouest Acute Leukemia (FHU GOAL), 49933 Angers, France; 5 Centre de Recherche en Cancérologie et Immunologie Nantes Angers (CRCINA), INSERM, University of Angers, 49933 Angers, France

**Keywords:** azacitidine, docosahexaenoic acid, eicosapentaenoic acid, PUFAylation, myelodysplastic syndromes, nanomedicine

## Abstract

5-Azacitidine, a cytidine analogue used as a hypomethylating agent, is one of the main drugs for the treatment of myelodysplastic syndromes (MDSs) and acute myeloid leukemia (AML) in the elderly. However, after administration, it exhibits several limitations, including restricted diffusion and cellular internalization due to its hydrophilicity, and a rapid enzymatic degradation by adenosine deaminase. The aim of this study was to improve the drug cell diffusion and protect it from metabolic degradation via the synthesis of amphiphilic prodrugs and their potential self-assembly. Azacitidine was conjugated to two different omega-3 fatty acids, eicosapentaenoic acid (EPA) and docosahexaenoic acid (DHA). The carboxylic acid group of the omega-3 fatty acids was effectively conjugated to the amine group of the azacitidine base, yielding two amphiphilic prodrugs. Nanoprecipitation of the obtained prodrugs was performed and self-assemblies were successfully obtained for both prodrugs, with a mean diameter of 190 nm, a polydispersity index below 0.2 and a positive zeta potential. The formation of self-assemblies was confirmed using pyrene as a fluorescent dye, and the critical aggregation concentrations were determined: 400 µM for AzaEPA and 688 µM for AzaDHA. Additionally, the stability of the obtained self-assemblies was studied and after 5 days their final stable arrangement was reached. Additionally, cryo-TEM revealed that the self-assemblies attain a multilamellar vesicle supramolecular structure. Moreover, the obtained self-assemblies presented promising cytotoxicity on a leukemia human cell line, having a low IC_50_ value, comparable to that of free azacitidine.

## 1. Introduction

**Figure 1:**
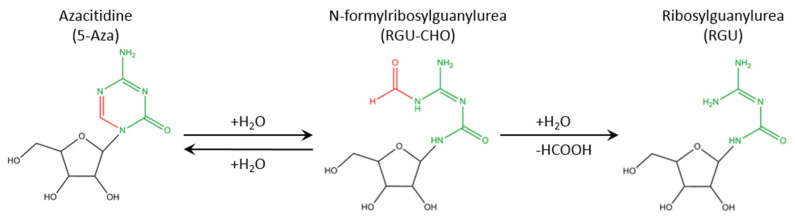
Hydrolysis of azacitidine. In the presence of water, a fast and reversible hydrolysis occurs, producing N-formylribosylguanylurea, followed by a second slow irreversible hydrolysis, producing ribosylguanylurea.

Myelodysplastic syndromes (MDSs) are clonal hematopoietic stem cell disorders characterized by evident morphological dysplasia (abnormal cell differentiation), showing variable degrees of cytopenia (decreased blood cell counts), with frequent progression into acute myeloid leukemia [1]. 5-azacitidine (Figure 1) is one of the drugs approved for the treatment of (i) higher-risk MDS (the advanced form of MDS) as it prolongs the overall survival [2,3]. (ii) It can also be used in lower-risk MDS patients following the failure of conventional approaches [4,5], (iii) in the case of delays for patients eligible for hematopoietic stem cell transplantation procedures and in addition as post-procedure relapse prevention [6,7,8], as well as, (iv) as a first-line treatment for AML [9,10,11]. Azacitidine is nowadays the backbone of therapy association in MDS trials and its use in association with venetoclax is the new standard for unfit AML patients.

Upon the entry of this DNA-hypomethylating cytidine analogue, via the action of human equilibrative nucleoside transporter 1 (hENT1), azacitidine undergoes successive phosphorylation steps via the regular nucleic acid kinases, to reach its final tri-phosphorylated form, allowing its integration into the DNA. It acts as well on the DNA methyltransferase, causing permanent inhibition of the enzyme, leading to the alteration of gene expression by decreasing the methylation levels of the cytosine, thus impacting the DNA epigenetic status and allowing the reactivation of tumor suppressor genes [12,13].

Clearly, azacitidine is a crucial weapon in the arsenal against these two diseases, and coupled with a lack of other satisfying alternatives, this further demonstrates its significance. However, only 50% of MDS patients respond to azacitidine (Fenaux et al., *The Lancet Oncology* 2009) and the median duration of response is around 24 months. A grim prognosis awaits patients upon the failure of azacitidine, with a decreased median overall survival to 5–7.5 months [14,15]. This failure can be either a primary failure (lack of any response to azacitidine), a secondary failure (disease progressed after a first response, serum drug level is low) or a toxicity failure (azacitidine treatment was terminated due to severe side effects) [15,16]. A part of these failures can be attributed to the chemical and pharmacological properties of this drug, as azacitidine is sensitive to water, undergoing rapid and reversible hydrolysis, producing N-formylribosylguanylurea, followed by its irreversible hydrolysis to ribosylguanylurea (Figure 1) [17,18], which explains its short half-life after administration. Additionally, because of its hydrophilic nature, poor cell internalization has been observed, which is a common problem for most hydrophilic drugs. Finally, the presence of nucleoside deaminase in the blood further decreases the half-life of this molecule and allows its rapid degradation and elimination [18,19,20,21,22].

In this study we aimed to counter these varied shortcomings with a dual approach—first, through the synthesis of an azacytidine-based prodrug via its conjugation to a fatty acid at the amine group of azacitidine, and second, by pushing the obtained prodrug towards self-assemblies. The obtained amphiphilic prodrug would have an enhanced entry into the cells owing to the similar nature of the cell lipid bilayer membrane [23,24], with a specificity to the affected cells owing to their overexpression of cathepsin-B, an enzyme that can cleave the conjugating amide bond, specifically releasing the free azacitidine [25,26,27]. Additionally, the prodrug-based self-assemblies would be able to further protect the azacitidine from degradation by deaminases, increasing its circulation time and thus reducing the required dose to achieve an equal response [12].

The choice of fatty acids to be conjugated to azacitidine was dictated by the presence of at least one double bond; thus, docosahexaenoic acid (DHA) and eicosapentaenoic acid (EPA) were chosen. Indeed, the presence of double bonds on the lipophilic part is an essential asset in order to obtain spontaneous self-assemblies in water, thanks to π–π stacking interactions [28,29,30,31,32].

Moreover, EPA and DHA have shown promising anticancer activity in various studies. DHA and EPA inhibited the growth of AML cell lines and induced cell death via oxidative stress pathways. Furthermore, they showed a synergistic effect after the combination of the fatty acids with cytarabine, a previously used hypomethylating agent [33]. Additional studies on DHA and EPA identified different pathways that these fatty acids utilize to impede cancer progression [34,35,36,37]. In vivo studies have also demonstrated that omega 3 fatty acids were able to reduce the number of abnormal progenitor cells and push them towards myeloid differentiation. The omega 3 and 6 fatty acids of the mouse diet were controlled, the omega 3 ones being in a higher proportion, which led to a decrease in the number of myeloid progenitor cells, while increasing differentiation without affecting peripheral white blood cell numbers, showing their promise in the treatment of MDS and AML [38].

The process of conjugating fatty acids to nucleosides and their analogues in order to combat such shortcomings is a well-investigated strategy. Indeed, this process has been pioneered by Prof. Couvreur and his team, with the use of squalene and its derivatives, which were conjugated to different nucleoside analogs in a process termed “squalenoylation”. This method was reproduced with other fatty acids, mainly poly-unsaturated ones, and termed “PUFAylation” [39,40,41,42,43].

Herein, we report on the synthesis of conjugating azacitidine to EPA and DHA, their nanoprecipitation of the obtained prodrugs and the characteristics of these self-assemblies.

## 2. Results and Discussion

**Figure 2:**
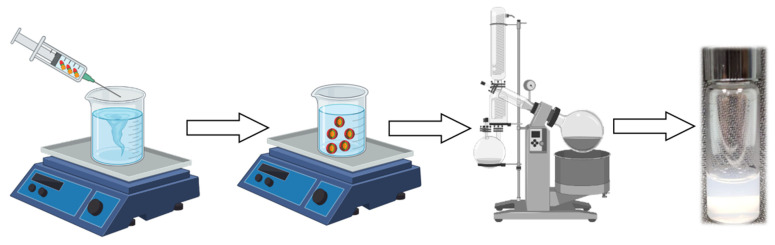
Formulation of self-assemblies via nanoprecipitation. The conjugates are dissolved in an organic solvent, here acetone, then added drop-wise to a stirring aqueous medium, allowing for the spontaneous formation of self-assemblies. The organic solvent is then evaporated using a rotary evaporator, thus yielding an opalescent aqueous suspension.

By conjugating azacitidine to EPA and DHA, two different prodrugs were obtained—an azacitidine-docosahexaenoic acid conjugate (N^4^-azacitidine DHA, AzaDHA) and an azacitidine-eicosapentaenoic acid conjugate (N^4^-azacitidine EPA, AzaEPA). These conjugates were obtained via a straightforward direct conjugation of the acid and amine moiety in the presence of ethyl chloroformate. The nanoprecipitation of the obtained prodrugs was performed and self-assemblies were successfully obtained (Figure 2) for both prodrugs with a diameter of ~190 nm, a polydispersity index below 0.2 and a positive zeta potential. Self-assemblies were verified by using pyrene as a fluorescent dye to determine the critical aggregation concentration. Furthermore, the formed self-assemblies needed 5 days to reach their final stable organization.

**Figure 3:**
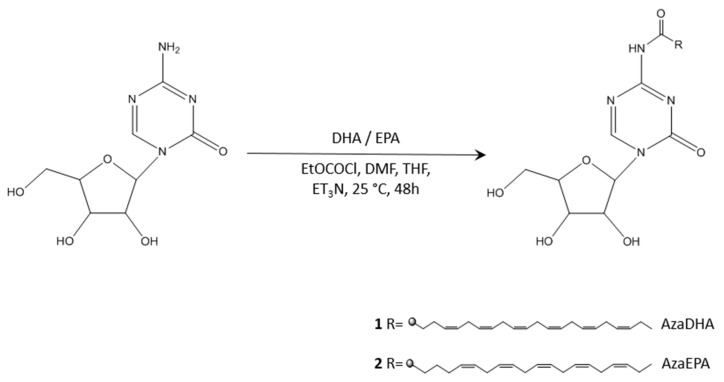
Synthesis of N4-azacitidine DHA (AzaDHA, 1) and N4-azacitidine EPA (AzaEPA, 2).

Nucleoside analogue derivatives that are coupled at the 4-(N)-position to various molecules exhibit enhanced metabolic stability in plasma, owing to the occupation of the deamination site, thus protecting these analogues from irreversible hydrolytic degradation [12,32,44,45]. As a consequence of their hydrophobic nature, both DHA and EPA can act as the scaffold in the synthesis of amphiphilic prodrugs [46,47]. In this study, azacitidine was covalently bound to either EPA or DHA, to form N^4^-azacitidine DHA (AzaDHA) and N^4^-azacitidine EPA (AzaEPA) amphiphilic prodrug conjugates through amide linkage (Figure 3). The AzaEPA and AzaDHA prodrugs were then purified via semi-preparative reversed phase high-performance liquid chromatography (RP-HPLC) with a final mean yield of ~10% and a purity of 92% for AzaEPA; AzaDHA was obtained with a mean yield of ~20% and a purity of 97%. The chemical structure of the obtained conjugates was verified via ^1^H and ^13^C NMR spectroscopy with characteristic chemical shifts. Mass spectrometry (Figures S1 and S2) confirmed these results with 528.48 and 554.21 g.mol^−1^ values obtained for AzaEPA and AzaDHA, respectively. The method to determine purity is described in Figures S3–S5.

**Figure 4:**
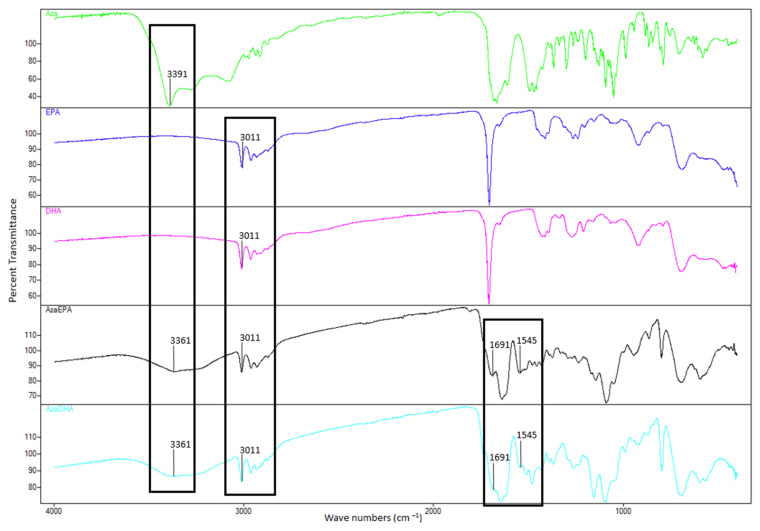
Fourier-transform infrared spectroscopy (FTIR) curves and wave numbers of interest between 4000 cm^−1^ and 600 cm^−1^ of azacitidine (green), EPA (purple), DHA (pink), AzaEPA (black) and AzaDHA (blue).

Fourier transform infrared spectroscopy (FTIR, Figure 4) confirmed the successful amide conjugation: the NH_2_ stretch of azacitidine at 3391 cm^−1^ disappeared after conjugation and was replaced with an NH stretch in both AzaEPA and AzaDHA conjugates at 3361 cm^−1^. Additionally, the SP^2^ C-H stretches belonging to the fatty acid chains appeared at 3011 cm^−1^ in both conjugates. Finally, the carboxylic group C=O stretches at 1691 cm^−1^ and the fatty acid double bond C=C stretches appeared at 1545 cm^−1^. All the mentioned changes are thus indicative of the success of the conjugation in producing the two prodrugs.

Similarly, elemental analysis further confirmed the successful conjugation as the obtained elemental percentages of C, H and N atoms closely matched the theoretically calculated ones.

As azacitidine is conjugated to two different polyunsaturated fatty acids, the potential process of self-assembly is termed “PUFAylation”. Though this term was recently introduced by Liming Wu et al. [45], the concept of conjugating a polyunsaturated fatty acid (PUFA) to a nucleoside, leading to the spontaneous self-assembly of the created prodrug, has been tackled before. Certainly, this innovative strategy was established by Couvreur and colleagues, with squalene derivatives (a cholesterol precursor) conjugated to nucleoside analogs in a process they termed “Squalenoylation” [48]. What is more, “PUFAylation”, while permitting this conjugate to self-assemble in water, does not require any additional excipient, providing an advantage over traditional nanomedicines such as liposomes or polymeric nanoparticles, thus improving the drug tolerability in animals and demonstrating exceptional effectiveness, with unbeatable drug loading results. Moreover, this method produces prodrugs with adequate amphiphilicity, allowing for an enhanced in vivo antitumor efficacy compared to the parent drug. Moreover, most of these “PUFAs” are essential for biological functions and are plentiful in the body, meaning that it may reduce toxicities stemming from the use of adjuvants, and finally the π-π stacking interaction between PUFAs would increase the stability of the self-assemblies [49]. Based on all this information, PUFA technology is an immensely encouraging strategy for the manufacturing of prodrugs and their derived self-assembled nanomedicines [50,51,52,53].

An alternative approach (Figure S6) was first explored to achieve the conjugation based on the work of Gaudin et al. [54]. To start, the highly reactive alcohol groups of the sugar ring were protected via the action of a silylating agent, TBDMSCl (tert-Butyldimethylsilyl chloride) in this case. Following this, an amide conjugation will allow the desired fatty acids to be linked to azacitidine, via the action of ethyl chloroformate as a conjugating agent. Finally, the protection groups of the obtained silylated prodrug were removed using TBAF (tetra-n-butylammonium fluoride), a commonly utilized deprotecting agent. The process was successful and the prodrug was verified using mass spectra (Figures S12 and S13). However, the purification of the product following de-silylation was hindered and the direct conjugation method described initially (Figure 3) was developed to obtain the prodrugs. The protection approach is fully described in the Supplementary Data.

**Figure 5:**
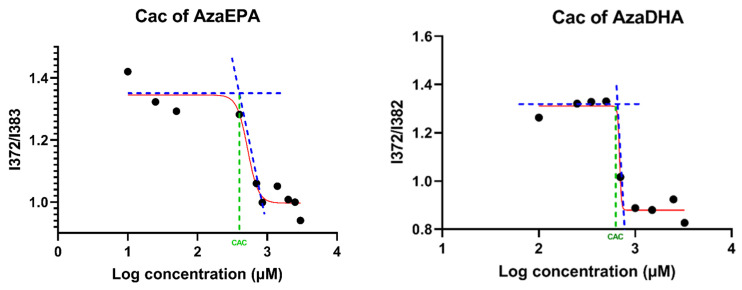
Boltzmann-type sigmoid data (red) obtained for the AzaEPA and AzaDHA suspensions in water, showing the CAC value, corresponding to the first sharp decrease point. Graph tangents (blue) are plotted, the tangent’s intersection with the graph (green) determines the CAC.

To study the potential self-organization of the AzaEPA and AzaDHA prodrugs, the fluorescent dye pyrene method was used. Briefly, the pyrene fluorescence spectra, and specifically the emission band intensities at 372 nm and 383 nm are different depending upon whether the pyrene is in a polar or apolar environment: the ratio of these intensities will be higher than 1 in a polar environment such as water and will be below 1 in a hydrophobic core. Thus, in plotting the I_372_/I_383_ ratio as a function of the prodrug concentration, if a self-assembly phenomenon occurs, a sigmoidal curve should fit the experimental points, and the CAC can be measured. For both prodrugs, a sigmoidal curve was obtained, proving the successful self-organization of the AzaEPA and AzaDHA. The CAC were determined as the first sharp decrease point [55,56] (Figure 5): 400 µM for AzaEPA and 688 µM for AzaDHA, respectively.

The concentration of 2 mg/mL, corresponding to 3.78 mM in AzaEPA and to 3.6 mM in AzaDHA, was then chosen to study the formulation of both amphiphilic conjugates via nanoprecipitation.

The AzaEPA and AzaDHA conjugates were dissolved in acetone, then this organic phase was added drop-wise to MiliQ deionized water, leading to an aqueous suspension with a characteristic opalescence, an indicator of a suspension of nanoparticles [57]. The organic phase was then evaporated using a rotary evaporator.

Interestingly, diverse behaviors were observed after evaporation based on the water-to-acetone ratio at higher final concentrations (≥4 mg/mL). At these high concentrations, when the volume ratio of water:acetone was of 4 (*v*/*v*), visually detectable aggregates were observed in the suspension, whereas at a volume ratio of 5, a homogeneous suspension of nanoparticles was obtained. The influence of the experimental parameters on the obtained self-assemblies via nanoprecipitation is well-known, notably the water:organic solvent ratio and the concentration in solute. Indeed, to be in the Ouzo zone during the formulation process, in order to obtain stable nanoobjects, a highly diluted solution with a large volume of water has to be used.

The hydrodynamic diameter of these self-assembled nanoparticles was characterized by means of dynamic light scattering (DLS). Values of 194.5 nm ± 0.12% for AzaEPA and 185 nm ± 0.95% for AzaDHA were obtained, with a low polydispersity index < 0.2 (day 5, Table 1, Figures S16 and S17). These results clearly verify that the PUFAylated azacitidine derivatives were able to form self-assembled nanoparticles devoid of the need for any excipients, with a 100% encapsulation efficacy and a drug loading of 44% for AzaDHA and 46% for AzaEPA, showing an improvement compared to conventional nanocarriers such as liposomes [58]. Regarding electrophoretic mobility, a potential zeta of 16.1 mV ± 8.54% for AzaEPA and 18 mV ± 6.74% for AzaDHA were obtained after 1 day of formulation.

**Figure 6:**
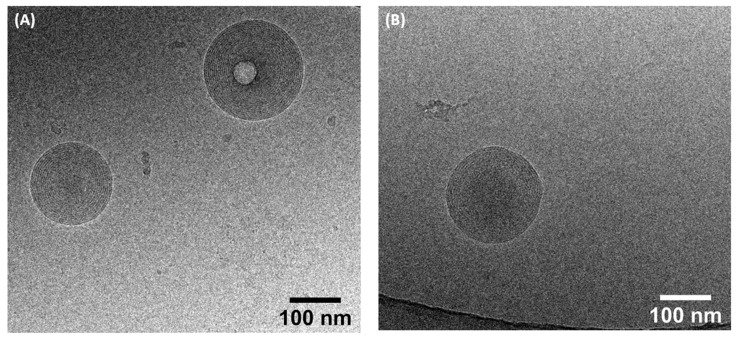
Cryo-TEM images of the formed self-assemblies: (**A**) AzaEPA, (**B**) AzaDHA.

Additionally, cryo-transmission electron microscopy (cryo-TEM) analysis was conducted to detect the morphology of the self-assemblies. The results (Figure 6) showed that the AzaDHA and AzaEPA conjugates self-assembled into multilamellar vesicles with a mean diameter around 180 nm for AzaDHA self-assemblies, whereas the AzaEPA conjugate nanoparticles had a mean diameter around 190 nm.

The physico-chemical parameters’ stability was studied and followed by DLS and by electrophoretic mobility. Interestingly, despite a PDI that was always below 0.2, illustrating a monodisperse suspension, fluctuating zeta potential values were observed until attaining their final stable form at day 5 (Table 1). This observation could be attributed to the supramolecular organization of these amphiphilic prodrugs, reaching their equilibrium after 5 days. The positive surface charge would mean that cationic amines would be in the surface of the structure. Moreover, nanoassemblies have a diameter of around 200 nm, that cannot reflect a simple micelle organization (the obtained prodrugs have a length of 17.4 Å (1.74 nm) for AzaDHA and 21.2 Å (2.21 nm) for AzaEPA, implying micelles of around 4–5 nm in diameter). Therefore, it can be hypothesized that these self-assemblies are organizing into a larger, more complex supramolecular structure. This phenomenon has already been observed in similar research carried out by Lepeltier et al., in which squalene-based nucleolipid conjugates attained different complex supramolecular structures depending on the site of conjugation rather than forming small micelles; additionally, the difference in the shape of the formed supramolecular structures led to a difference in their pharmacological activities [32,50,57]. Therefore, the obtained AzaEPA and AzaDHA nanoassemblies form complex molecular structures that require a few days to attain their final stable structure, with a positive zeta potential that could increase the cellular internalization by facilitating a charge-based interaction with the negatively-charged cell membrane surface [59].

**Figure 7:**
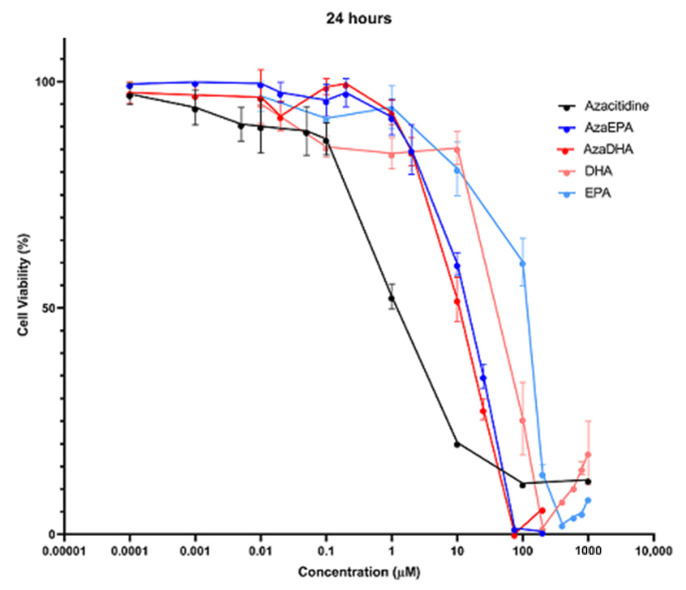
Cytotoxicity studies of the self-assemblies compared to the free azacitidine and fatty acids after 24 h of treatment.

A preliminary in vitro study was conducted on HL-60 cells, to determine the cytotoxicity of the AzaDHA and AzaEPA self-assembled conjugates and compare them to the azacitidine and the fatty acids. An MTT assay was performed after 6 (Figure S14), 24 (Figure 7) and 48 (Figure S15) hours of treatment incubation times, with various concentrations, and IC_50_ values were determined (Table 2).

The cytotoxicities at 6, 24 and 48 h reflected the same results: well-defined dose-response curves showing that azacitidine was the most cytotoxic of the molecules tested, followed by AzaDHA and AzaEPA, which were 10-fold less cytotoxic than the parent molecule. Finally, the free fatty acids appeared to have the least cytotoxic effect. Interestingly, similarly to the free fatty acids—where DHA had an increased cytotoxic effect compared to EPA—the cytotoxic effect of the DHA-based self-assemblies was better than that of the EPA-based ones. The half-maximal inhibitory concentrations (Table 2) further verified these observations, as the same trend was observed at the three time points.

As expected, the cytotoxicity of the AzaEPA and AzaDHA self-assemblies was weaker than that of free azacitidine. This difference in cytotoxicity is often observed for prodrugs [60,61]. Certainly, the azacitidine molecule must be first cleaved from the fatty acid conjugates to regain its pharmacological activity. In vitro, even though HL-60 cells produce the cathepsin B enzyme, which is able to release azacitidine from its conjugation [62,63,64], this release is slow and progressive. For a prodrug, it is known that the biological efficacy has to be determined in vivo, to see all its potential. The coupling of nucleoside analogues with fatty acids was shown to reduce the deamination and increase the in vivo drug half-life, modifying its pharmacokinetics and biodistribution [41,61,65,66]. Additionally, azacitidine in its clinical application is used as a hypomethylating agent, not as a cytotoxic agent, and the dose needed is less than the IC_50_; thus, these results reflect positively on the success of the studied self-assemblies. Furthermore, the need for cathepsin B to release the azacitidine will increase its specificity and decrease the toxic side effects on healthy tissues [62,63]. In the cases of the envisioned in vivo studies, following the traditional clinical administration route of azacitidine, the prodrug self-assemblies should be administered in a similar method of subcutaneous or intravenous injections.

## 3. Materials and Methods

### 3.1. Materials

Dimethylformamide, methanol, tetrahydrofuran, trimethylamine, ethyl chloroformate, dichloromethane, sodium dihydrogen phosphate, magnesium sulfate and acetonitrile were obtained from Fisher (Thermo Fisher Scientific, Strasbourg, Grand Est, France). Eicosapentaenoic acid and docosahexaenoic acid were obtained from Combiblocks (San Diego, CA, USA). 5-azacitidine was obtained from TCI chemicals (Tokyo, Kanto, Japan). Deionized water was obtained from a Milli-Q plus system (Merck-Millipore, Saint-Quentin-en-Yvelines, Île-de-France, France). Acetone and pyrene were obtained from Sigma-Aldrich (Saint-Quentin-Fallavier, Auvergne-Rhône-Alpes, France).

### 3.2. Synthesis and Purification

One equivalent of either omega-3 fatty acid (5Z,8Z,11Z,14Z,17Z)-eicosa-5,8,11,14,17-pentenoic acid (Eicosapentaenoic acid, EPA) or (4Z,7Z,10Z,13Z,16Z,19Z)-docosa-4,7,10,13,16,19-hexaenoic acid (Docosahexaenoic acid, DHA) was mixed with 2 equivalents of trimethylamine in 3 mL of dry tetrahydrofuran (THF) for 20 min at room temperature. The flask was cooled down to −10 °C using an acetone ice bath and mixed for an additional 10 min; 2 equivalents of ethyl chloroformate in 3 mL THF were then added drop-wise to the mixture and allowed to mix for 10 min. Then, 100 mg of 4-amino-1-[(2R,3R,4S,5R)-3,4-dihydroxy-5-(hydroxymethyl)oxolan-2-yl]-1,3,5-triazin-2-one (5-azacitidine) in 9 mL of dimethylformamide (DMF) were added drop-wise and allowed to mix for 10 min. The mixture was then removed for the acetone ice bath to reach room temperature and stirred for 48 h. The whole procedure was conducted under argon.

The mixture was then dried on a rotary evaporator at 40 °C. Aqueous 0.02 M sodium dihydrogen phosphate (NaH_2_PO_4_) was added and the mixture was extracted with dichloromethane. The organic layer was then washed with brine, dried on magnesium sulfate (MgSO_4_) and concentrated in a vacuum.

The obtained oil was then purified by means of semi-preparative reversed phase high-performance liquid chromatography (RP-HPLC) using a (Guyancourt, Île-de-France, France) instrument. Purification was performed at room temperature using an XBridge BEH C18 OBD Prep Column, 130 Å pore size, 5 µm particles, 30 mm × 250 mm. Eluent (A) was acetonitrile (ACN), whereas eluent (B) was MiliQ H_2_O. A gradient elution was used with a flow rate of 37 mL/min and an injection volume of 1.5 mL. Peaks were detected at a wavelength of 214 nm (amide bond) and 240 nm (azacitidine). The crude oil was solubilized in 90% (A) and 10% (B) at a concentration of 10 mg/mL. The sample was vortexed, sonicated and filtered on a 0.22 μm Millex-LG filter (Merck-Millipore, Saint-Quentin-en-Yvelines, Île-de-France, France) prior to injection. The collected purified conjugate was then concentrated in a vacuum and lyophilized to obtain a white powder (AzaDHA: 45 mg yield 20% ± 2.1%, AzaEPA: 21 mg yield 10% ± 1.4%).

### 3.3. ^1^H and ^13^C NMR

^1^H NMR and ^13^C NMR spectra were recorded in deuterated dimethyl sulfoxide (DMSO-d6) at 400 MHz for the ^1^H NMR, and at 125 MHZ for the ^13^C NMR with a Bruker 500MHz AVANCE III HD spectrometer (Wissembourg, Grand Est, France) equilibrated at 25 °C, at the SFR Matrix of the University of Angers. Spectra were analyzed using the software MestReNova^®^ version: 12.0.0-20080.

AzaEPA: ^1^H NMR (499 MHz, DMSO-d6) δ 8.55 (s, 1H), 7.66–7.58 (m, 1H), 5.71 (d, J = 5.8 Hz, 1H), 5.40–5.28 (m, 10H), 5.17–5.04 (m, 1H), 4.64–4.49 (m, 1H), 4.42 (q, J = 5.5 Hz, 1H), 4.34–4.18 (m, 1H), 4.09–4.00 (m, 1H), 3.89 (ddt, J = 27.4, 6.0, 3.0 Hz, 1H), 3.65 (tdd, J = 11.1, 5.9, 3.2 Hz, 1H), 3.61–3.53 (m, 1H), 2.85–2.76 (m, 8H), 2.44–2.35 (m, 2H), 2.11–2.01 (m, 4H), 1.60 (ddt, J = 14.5, 10.4, 4.8 Hz, 2H), 0.92 (tt, J = 7.6, 1.9 Hz, 3H). ^13^C NMR (125 MHz, DMSO) δ 172.11, 165.68, 157.72, 153.04, 131.55, 128.11, 128.02, 127.89, 127.70, 126.94, 90.35, 84.05, 74.07, 68.01, 59.28, 36.42, 25.97, 25.20, 20.03, 14.11.

AzaDHA: ^1^H NMR (499 MHz, DMSO-d6) δ 8.61 (d, J = 11.4 Hz, 1H), 7.83 (s, 1H), 5.71 (d, J = 5.2 Hz, 1H), 5.42–5.24 (m, 12H), 4.42 (t, J = 5.3 Hz, 1H), 4.29 (q, J = 6.1, 5.7 Hz, 1H), 4.21–4.15 (m, 1H), 4.07 (dd, J = 5.1, 2.4 Hz, 2H), 3.73–3.64 (m, 2H), 3.59–3.54 (m, 1H), 2.81 (dq, J = 21.7, 6.4, 5.7 Hz, 10H), 2.43 (d, J = 7.3 Hz, 2H), 2.33 (q, J = 7.8, 7.2 Hz, 2H), 2.04 (td, J = 7.4, 1.5 Hz, 2H), 0.92 (td, J = 7.6, 1.4 Hz, 3H). ^13^C NMR (125 MHz, DMSO) δ 172.10, 157.74, 156.47, 153.12, 131.55, 127.90, 126.94, 90.35, 88.83, 74.07, 71.94, 68.00, 36.87, 25.20, 22.14, 20.03, 14.11.

### 3.4. Elemental Analysis

Elemental analyses of C and H were conducted by Thermo Scientific (Strasbourg, Grand Est, France)—Elemental analyzer FLASH 2000 in CHNS mode at the SFR Matrix of the University of Angers.

AzaEPA, elemental analysis calculated (%) for C_28_H_40_N_4_O_6_: C 63.62, H 7.63, N 10.6; found: C 61.66, H 7.63, N 9.67. AzaDHA, elemental analysis calculated (%) for C_30_H_42_N_4_O_6_: C 64.96, H 7.63, N 10.1; found: C 63.2, H 7.71, N 9.31.

### 3.5. Fourier-Transform Infrared Spectroscopy

Fourier-transform infrared spectroscopy (FTIR) spectra were recorded on a ThermoFisher Scientific (Strasbourg, Grand Est, France) Nicolet iS5 FTIR, with an iD7 ATR Diamond crystal accessory, for powder analysis. The spectra were obtained applying 16 scans per spectrum after 16 background scans and were analyzed in the frequency range of 4000–600 cm^−1^.

### 3.6. Mass Spectrometry

The conjugates were dissolved in acetonitrile + 0.1% formic acid at a concentration of 50 µg/mL. The solution was directly infused at 10 µL/min into a Quattro Micro^®^ triple quadrupole mass spectrometer (Waters). Prior to infusion, the sample was vortexed, sonicated and filtered on a 0.22 μm Millex-LG filter (Merck-Millipore, Saint-Quentin-en-Yvelines, Île-de-France, France). Ionization was achieved using an electrospray technique in positive-ion mode. The mass spectrometer was operated in multiple-reaction-monitoring mode. The entire system was controlled using Masslynx^®^ software version 4.1 (Waters).

### 3.7. Critical Aggregation Concentration (CAC)

The CAC of the AzaEPA and AzaDHA suspensions was determined using pyrene as a fluorescent probe. Briefly, 6 μL of pyrene stock solution in acetone (50 μM) was added into tubes. Then, acetone in tubes was evaporated in dark condition. The different suspensions with a concentration ranging from 10 to 3000 μM were added into tubes and mixed overnight at 37 °C. The final concentration of pyrene was 1 μM. After 30 min of equilibration at room temperature, a fluorescence spectrophotometer (Fluoromax-4, Horiba, Kyoto, Japan) was used to measure the fluorescence intensities of pyrene at an excitation wavelength of 336 nm. The emission spectra were recorded in the range of 350–500 nm. The slit opening for the excitation was set at 1 nm and at 3 nm for the emission. Intensity ratios of pyrene at I_372_/I_382_ (I1/I3) were plotted against the log of the concentration.

### 3.8. Self-Assembly Formulation

AzaEPA and AzaDHA self-assemblies were prepared using the nanoprecipitation process. Briefly, AzaEPA or AzaDHA was dissolved in 0.25 mL of acetone (8 mg/mL) and added drop-wise under strong mechanical stirring to 1 mL of MiliQ water. The formation of the self-assemblies occurred spontaneously. The acetone was then completely evaporated using a rotary evaporator to obtain an aqueous suspension of self-assemblies with a final concentration 2 mg/mL in prodrugs.

### 3.9. Dynamic Light Scattering (DLS)

Hydrodynamic diameter by intensity distribution (Z-average size) and size polydispersity (PDI) were determined via dynamic light scattering on a Zetasizer^®^ Nano series DTS 1060 (Malvern Instruments S.A., Malvern, UK) at a scattering angle of 173°. Three consecutive measurements were performed for each sample. A good attenuator value (7 to 9) was obtained when suspending 20 μL of the self-assemblies in 1 mL of distilled water. The mean diameter for each preparation resulted from the average of three measurements of 60 s each. For zeta potential measurements, 20 μL of the self-assemblies were dissolved in 1 mL of 1mM NaCl before filling the measurement cell. The mean zeta potential for each preparation resulted from the average of three measurements in automatic mode. Size, PDI and zeta-potential are expressed as mean ± SD%.

### 3.10. Cryogenic Transmission Electron Microscopy (Cryo-TEM)

Cryo-TEM studies were accomplished with a Cryo-TEM (Tecnai^TM^ G2 Sphera, FEI, Hillsboro, OR, USA) at the Microscopy Rennes Imaging Center (Biogenouest, Rennes, France). A drop (4 μL) of the 4 mg/mL sample was deposited on the surface of a carbon-coated copper grid. This gird was held under controlled humidity and temperature conditions by tweezers on a guillotine device. A filter paper was then pressed against the sample to remove the excess liquid. Afterwards, the filter paper was removed, and the plunger was allowed to drop into the liquid ethane in order to vitrify the sample. The gird was then transferred to a cryo-holder. Observations were made at an accelerating voltage of 200 kV under a low electron dose. Analysis was performed with ImageJ software version 1.53e (NIH, Stapleton, NY, USA).

### 3.11. Cytotoxicity Assay

The cytotoxicity was determined with a colorimetric assay, using the succinate dehydrogenase activity of viable cells via the reduction of the yellow-colored tetrazolium salt, 3-(4,5-dimethylth-iazol-2-yl)-2,5-diphenyl tetrazolium bromide to a purple-colored formazan crystal (MTT assay). Briefly, HL-60 cells were plated in 96-well plates at densities of 100,000 cells/well. After 24 h, cells were treated for 6, 24 and 48 h with different concentrations of self-assemblies prepared from a 2 mg/mL mother solution of self-assemblies and diluted in media. Then, cells were incubated for 4 h with the MTT solution (0.5 mg/mL in PBS). The medium was removed and 0.1 M HCl-SDS solution (100 μL/well) was added to solubilize the formazan crystals. Samples were finally analyzed with absorbance detection at 570 nm on a plate reader (SpectraMax^®^ M2 System, Molecular Devices, San Jose, CA, USA). The control was performed with cells cultured in medium, without any treatment. Three independent experiments were conducted with triplicate samples. The half-maximal inhibitory concentration (IC_50_) was determined from the dose-response curve.

## 4. Conclusions

To conclude, an innovative platform was set up to transport a hydrophilic and a degradation-sensitive anti-cancer agent. The synthesis of two amphiphilic conjugates was successfully performed and verified via several methods, including ^1^H and ^13^C NMR, FTIR and elemental analysis. Their self-assembly in water was successful and confirmed, using the fluorescent probe pyrene method to determine the critical aggregation concentration. The formed nanoassemblies were then characterized, presenting a hydrodynamic diameter of 190 nm and a positive surface charge, and needing 5 days to attain a stable configuration, owing possibly to a complex supramolecular structure. This study highlights the importance of this method in protecting the vulnerable azacitidine drug, while increasing its bioavailability and offering improved drug-loading capabilities compared to traditional nanovectors. The precise supramolecular structure should be determined via synchrotron-based small-angle X-ray scattering. The obtained formulations had a low IC_50_ value, comparable to that of free azacitidine, allowing them to achieve close cytotoxicity. Additional in vitro studies on the Hl-60 cell line (human acute myeloid leukemia) will be conducted to compare the efficacy of the self-assemblies compared to the parent drug. Cell internalization studies will be performed, and the cell total DNA methylation profile will be studied via a LINE-1 methylation test to determine if azacitidine retains its mode of action after being conjugated to a fatty acid.

## Figures and Tables

**Table 1:** Stability of AzaEPA and AzaDHA self-assemblies over 7 days, reflected by the hydrodynamic diameter, zeta potential and polydispersity index (PDI). Data are expressed as mean ± SD%.

Sample	Days	Hydrodynamic Diameter (nm)	Zeta Potential (mV)	PDI	Attenuator
**AzaEPA**	1	235.8 ± 7.3%	16.1 ± 8.54%	0.121 ± 24%	7
	2	206.7 ± 1.57%	−9.89 ± 2.02%	0.169 ± 12.8%	7
	3	190.1 ± 1.37%	25.8 ± 2.33%	0.16 ± 16.6%	7
	4	190.5 ± 0.963%	25.9 ± 2.8%	0.149 ± 14.4%	7
	5	194.5 ± 0.119%	35.7 ± 7.12%	0.137 ± 22.7%	7
	6	213 ± 1.76%	33.2 ± 8.79%	0.15 ± 16.4%	7
	7	217 ± 2.89 %	35.8 ± 4.86%	0.184 ± 13%	7
**AzaDHA**	1	229.5 ± 0.575%	18 ± 6.74%	0.082 ± 23.9%	7
	2	232.5 ± 1.37%	21.9 ± 4.63%	0.094 ± 16.4%	7
	3	217.7 ± 0.87%	−2.63 ± 28.6%	0.075 ± 21.8%	7
	4	189.2 ± 0.242%	22.7 ± 6.68%	0.77 ± 47.2%	7
	5	185 ± 0.952%	17.8 ± 4.05%	0.055 ± 67.5%	7
	6	189.7 ± 0.777%	37.8 ± 2.97%	0.138 ± 14.3%	7
	7	182.5 ± 3.91%	33 ± 6.09%	0.155 ± 13.2%	7

**Table 2:** The half-maximal inhibitory concentration determined by an MTT assay on HL-60 cells, after treatment by azacitidine, fatty acids and self-assemblies at different time points.

IC50	Azacitidine	DHA	EPA	AzaDHA	AzaEPA
**6 h**	6.5 µM	100.2 µM	196.7 µM	27.5 µM	33.7 µM
**24 h**	1.0 µM	92.8 µM	115.9 µM	11.3 µM	16.6 µM
**48 h**	1.4 µM	44.5 µM	103.8 µM	10.2 µM	13.7 µM

## Data Availability

Data is contained within the article and supplementary files.

## Supplementary Materials

The following are available online at https://www.mdpi.com/article/10.3390/ph14121317/s1, Figure S1: Mass spectra of the azacitidine-EPA conjugate, Figure S2: Mass spectra of the azacitidine-DHA conjugate, Figure S3: Chromatogram of the azacitidine-EPA conjugate to determine its purity, Figure S4: Chromatogram of the azacitidine-EPA conjugate to determine its purity, Figure S5: Chromatogram of the azacitidine-DHA conjugate to determine its purity, Figure S6: The 3-step synthesis pathway for the synthesis of azacitidine-fatty acid conjugates, Figure S7: UPLC chromatogram of protected azacytidine, Figure S8: ^1^H NMR spectra of protected azacytidine, Figure S9: MS spectra of the protected azacytidine, Figure S10: Mass spectra of the protected azacitidine-EPA conjugate, Figure S11: Mass spectra of the protected azacitidine-DHA conjugate, Figure S12: Mass spectra of the azacitidine-EPA conjugate, Figure S13: Mass spectra of the azacitidine-DHA conjugate, Figure S14: Cytotoxicity studies on the HL-60 cell line of the self-assemblies compared to the free azacitidine and fatty acids at 6 h, via a MTT assay, Figure S15: Cytotoxicity studies on the HL-60 cell line of the self-assemblies compared to the free azacitidine and fatty acids at 48 h, via a MTT assay, Figure S16: Size distribution by intensity obtained by DLS for AzaDHA self-assemblies on day 7, Figure S17: Size distribution by intensity obtained by DLS for AzaEPA self-assemblies on day 7.
