# Supplementary material for: Azacitidine Omega-3 Self-Assemblies: Synthesis, Characterization, and Potent Applications for Myelodysplastic Syndromes
Source: Pharmaceuticals (Basel). 2021 Dec 17;14(12):1317. doi: 10.3390/ph14121317 (PMC8706301; doi:10.3390/ph14121317)
Supplement: Supplementary file 1 [file pharmaceuticals-14-01317-s001.zip › pharmaceuticals-1466063-supplementary.pdf]

Supplementary data

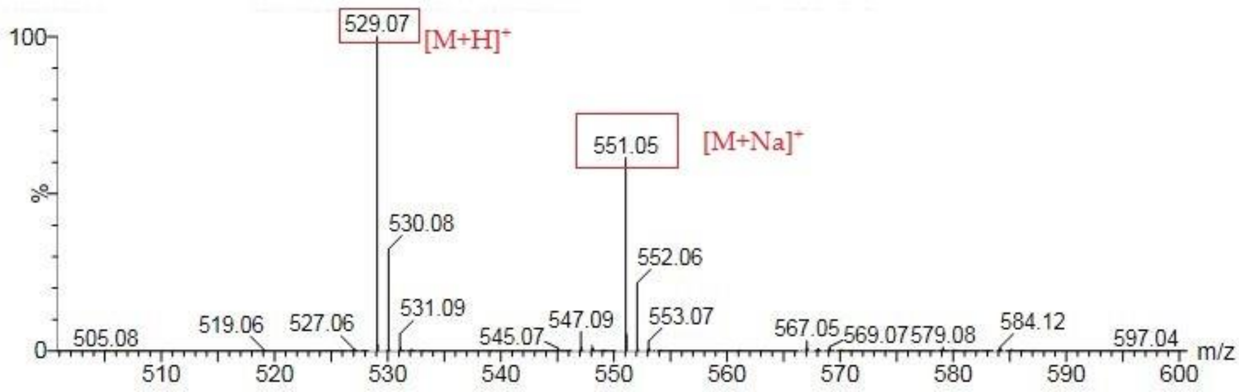

Figure S1: Mass spectra of the azacitidine-EPA conjugate.

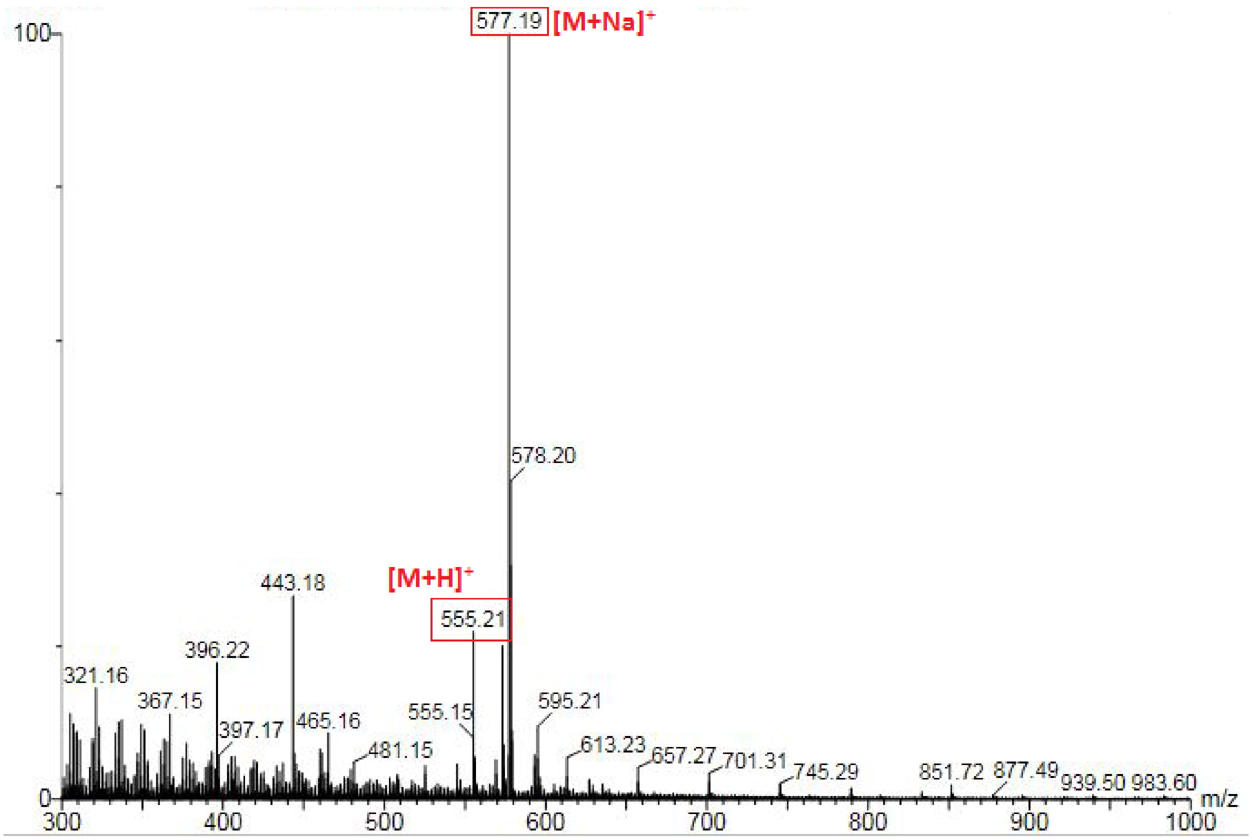

Figure S2: Mass spectra of the azacitidine-DHA conjugate.

A UPLC-UV method was developed to quantify the purity of the conjugates (**Figure S3**). A UPLC Acquity HClass Bio (Waters, France) consisting in a quaternary solvent manager, a sample manager, a photo diode array detector and a column manager was used. The system was controlled via Empower®3 software (Waters). The column used was an Acquity®UPLC BEH C18 50 x 2.1 mm, 1.7 µm (Waters). The mobile phase was composed of a mixture of acetonitrile and water (1:1). The purified product was dissolved in acetonitrile at a concentration of 100 µg/mL. Prior to injection, the sample was vortexed, sonicated and filtered on a 0.22 µm Millex-LG filter (Merck-Millipore, Germany). Flow rate was set to 0.2 mL/min and injection volume was set to 2 µL. The product was eluted in isocratic. Detection was fixed at 212 nm. Several peaks were observed, due to the use of an organic solvent and water, thus pushing the amphiphilic molecules to self-assemble, explaining the various retention times observed, but corresponding to the same conjugate.

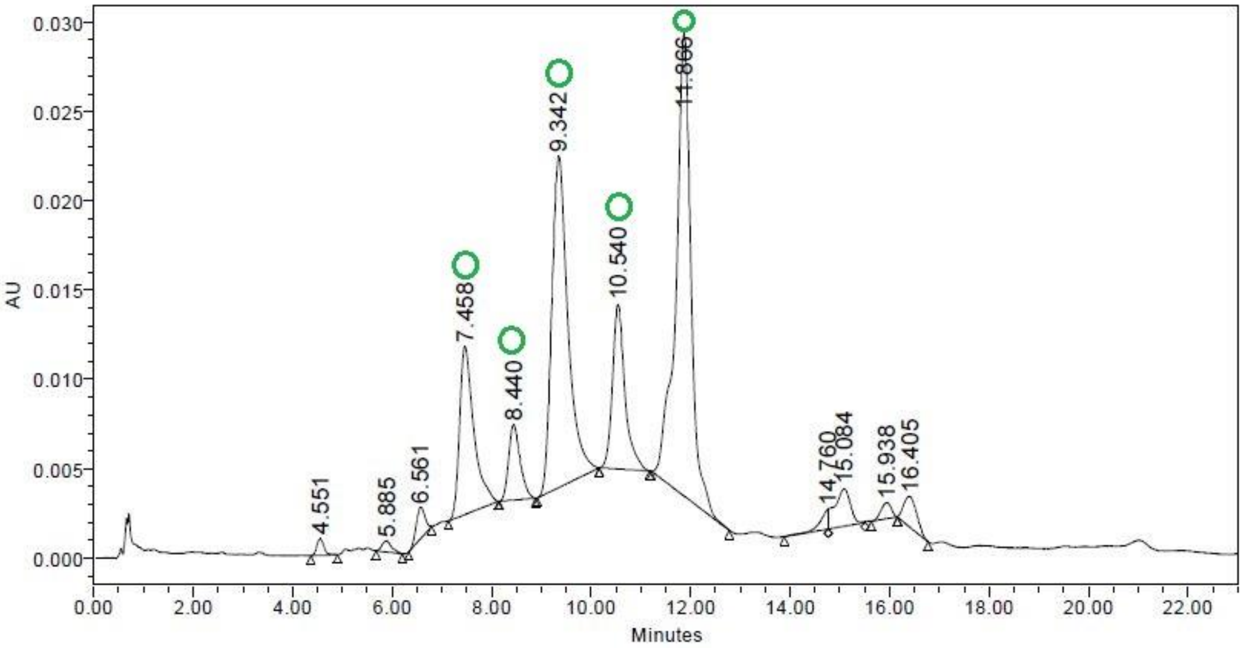

Figure S3: Chromatogram of the azacitidine-EPA conjugate to determine its purity. Peaks indicated by a green circle contain AzaEPA.

The UPLC-UV method was transferred to LC-MS using Waters software to achieve a similar chromatogram (**Figure S4 and Figure S5**).The LC-MS/MS method was developed on an Alliance® 2695 system (Waters) with a 150 × 2.0 mm, 5 µm Uptisphere C18 5ODB column (Interchim, France). The mobile phase was composed of a mixture of acetonitrile and water (1:1).

The product was eluted isocraticly. The purified product was dissolved in acetonitrile at a concentration of 1 mg/mL. The sample was vortexed, sonicated and filtered on a 0.22 µm Millex-LG filter (Merck, Germany). Flow rate was 0.2 mL/min and the injection volume was set at 5 µL. The total HPLC effluent was injected into a Quattro Micro®triple quadrupole mass spectrometer (Waters, France). Ionization was achieved using electrospray in positive ion mode in the m/z 200–1500 range (full scan acquisition). An option of cone ramp was used between 20 and 60 V to optimize the acquisition. The peaks of the MS chromatogram matched those of the UPLC, each was analyzed to determine the ones that contained the product, and finally the area under the curve of the UPLC chromatogram was taken into consideration to determine the purity of the products. AzaEPA purity was of 92 % while that of AzaDHA was of 97 %.

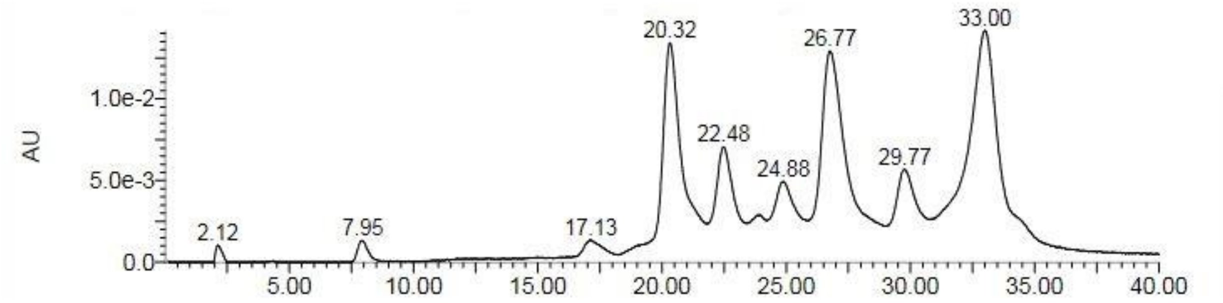

Figure S4: Chromatogram of the azacitidine-EPA conjugate to determine its purity.

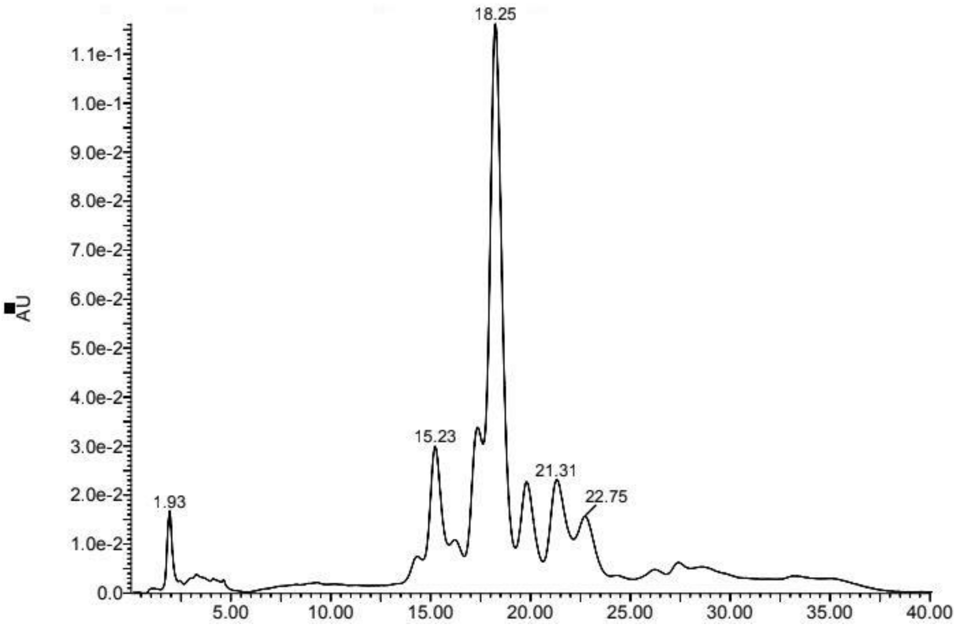

Figure S5: Chromatogram of the azacitidine-DHA conjugate to determine its purity

Alternative synthesis approach

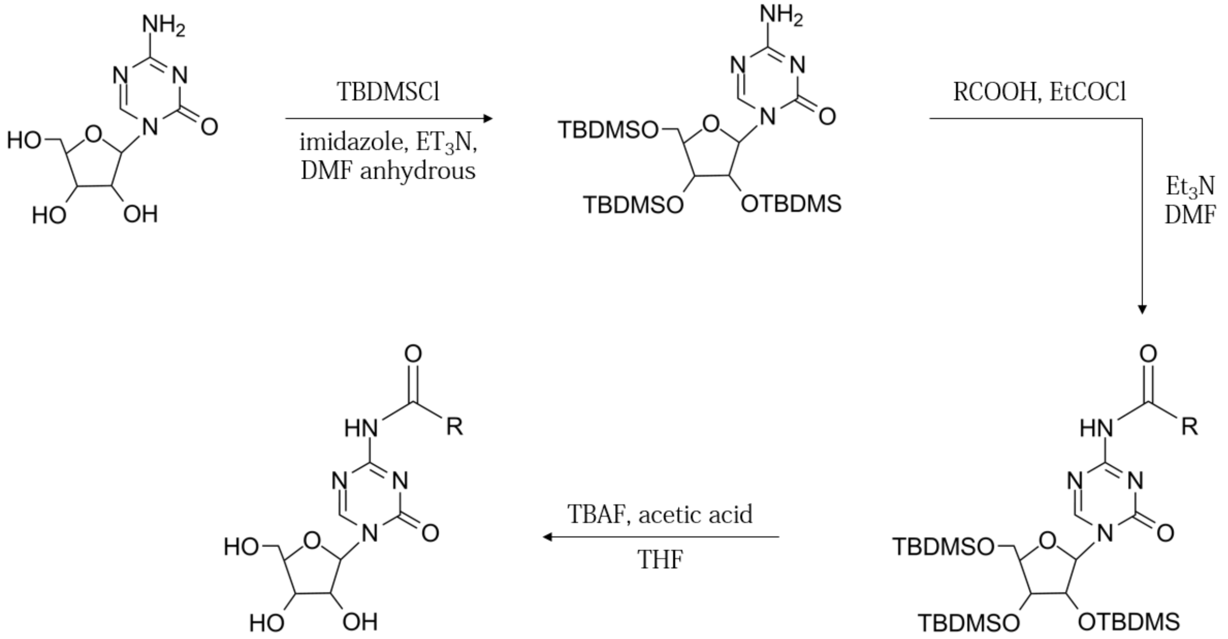

Figure S6: The 3-step synthesis pathway for the synthesis of azacitidine-fatty acid conjugates.

- **Protection of azacitidine**

To a solution of imidazole (15 equivalents) and tert-butyldimethylsilyl chloride (TBDMS, 12 equivalents) in anhydrous DMF (15 mL), stirred for 30 mins, 5-azacitidine (250 mg) in anhydrous DMF (20 mL) was added drop-wise, followed by the drop-wise addition of trimethylamine (5 equivalents) in anhydrous DMF (8 mL). The mixture was left to react at room temperature for 72 hours. The whole reaction was conducted under argon. The mixture was then concentrated using a rotary evaporator. The crude product was then purified by silica gel chromatography eluting with 4 % methanol in dichloromethane to give pure 4-amino-1-(3,4-bis((tert-butyldimethylsilyl)oxy)-5-(((tert-butyldimethylsilyl)oxy)methyl)tetrahydrofuran-2-yl)-1,3,5-triazin-2(1H)-on (tris-O-silylated azacitidine) as a colorless oil (565 mg,yield 94%, purity 90%). ^{1}H NMR (499 MHz, DMSO-d6) δ 8.46 (s, 1H), 7.61 – 7.53 (m, 2H), 5.69 (d, J = 3.8 Hz, 1H), 4.30 (t, J = 4.1 Hz, 1H), 4.14 (t, J = 4.5 Hz, 1H), 3.95 (q, J = 4.2, 3.7 Hz, 2H), 3.75 – 3.71 (m, 1H), 0.91 (s, 9H), 0.88 (s, 9H), 0.85 (d, J = 3.1 Hz, 9H), 0.14 – 0.02 (m, 18H).

A UPLC-UV method was developed to quantify the purity of the protected azacitidine (**Figure S7**). A UPLC Acquity HClass Bio (Waters, France) consisting of a quaternary solvent manager, a sample manager, a photo diode array detector and a column manager was used. The system was controlled via Empower®3 software (Waters). The column used was an Acquity®UPLC BEH C18 100 x 2.1 mm, 1.7 µm (Waters). The mobile phase was composed of a mixture of acetonitrile and methanol.

The purified product was dissolved in acetonitrile at a concentration of 0.5 mg/mL. Prior to injection, the sample was vortexed, sonicated and filtered using a 0.22 µm Millex-LG filter (Merck-Millipore, Germany). Flow rate was set to 0.6 mL/min and injection volume was set to 2 µL. The product was eluted via an isocratic flow. Detection was fixed at 241 nm to detect azacitidine. After using the area under the curve to analyze the obtained chromatogram, the purity of protected

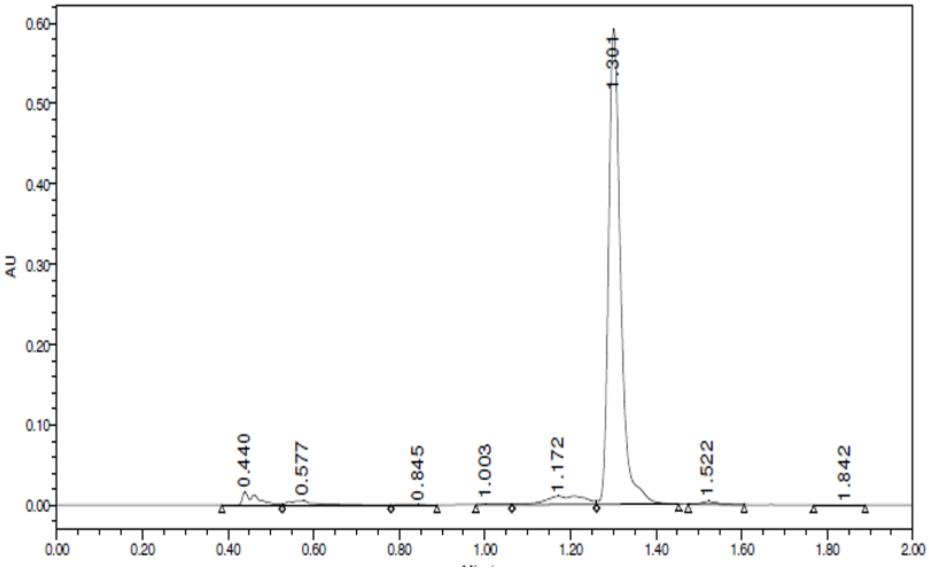

Peak Results

	Name	RT	Area	Height	Amount	Units
1		0.440	36745	16761		
2		0.577	22280	5537		
3		0.845	1797	473		
4		1.003	2361	908		
5		1.172	64684	10293		
6		1.301	1151289	592253		
7		1.522	9316	3534		
8		1.842	513	206		

Column : C18 BEH Acquity 2.1x100mm

Isocratic with 9:1 ACN:MeOH

Injection Volume : 2 µL

Run Time : 2 min

Flow Rate : 0.6 mL/min

λ : 241 nm

Figure S7: UPLC chromatogram of protected azacitidine.

azacitidine was determined of 90%: **Figure S7**.

^1H NMR spectrum of the protected azacitidine was recorded in deuterated dimethyl sulfoxide (DMSO-d6) at 400 MHz with a Bruker 500MHz AVANCE III HD spectrometer (Wissembourg, France) equilibrated at 25 °C, at the SFR Matrix of the University of Angers. Spectra were analyzed using the software MestReNova®. The methyl peaks of the protection groups were detected at 0.08 ppm and 0.85-0.91 ppm, the rest of the peaks conformed to those of azacitidine: **Figure S8**.

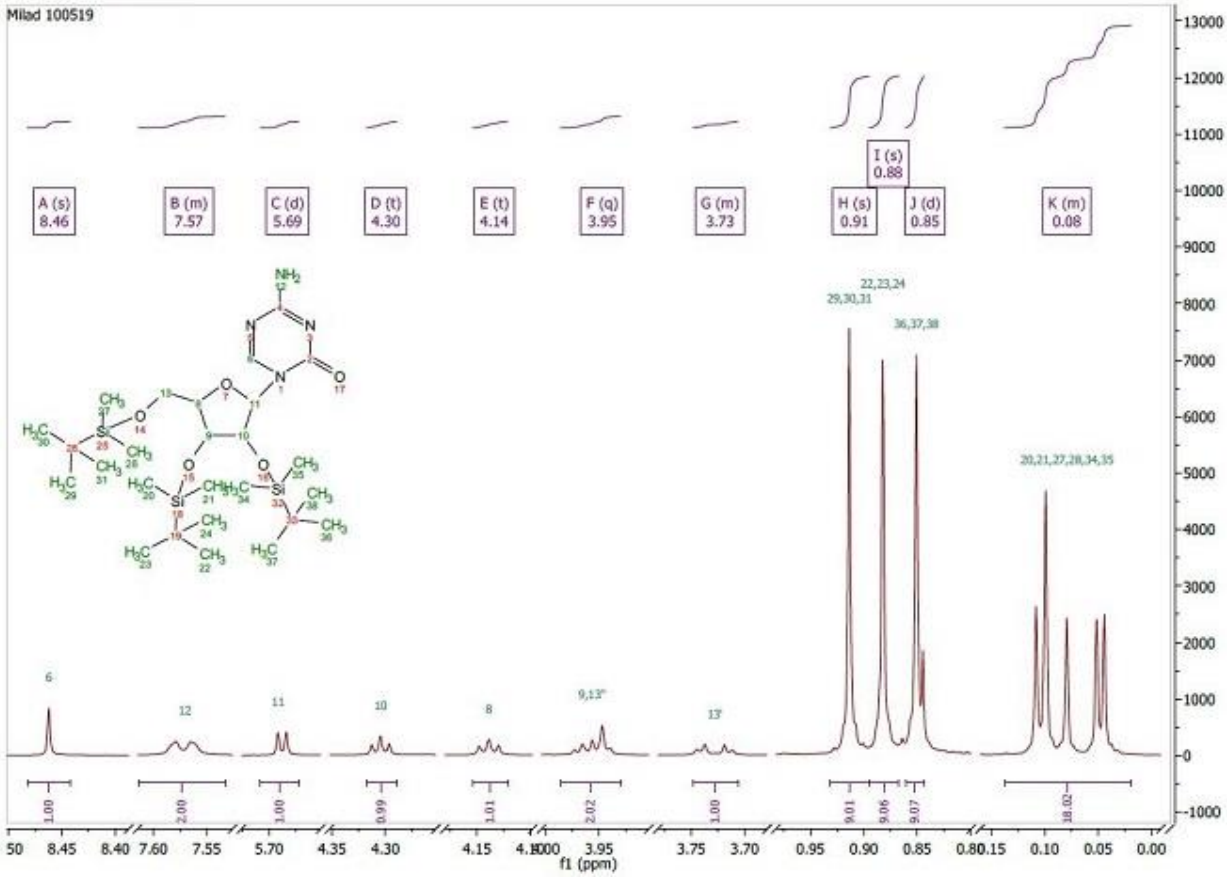

Figure S8: ^1H NMR spectra of protected azacitidine.

Mass spectrometry was used to further verify the obtained product. Protected azacitidine was dissolved in acetonitrile+0.1% formic acid at a concentration of 50 µg/mL. The solution was directly infused at 10 µL/min into a Quattro Micro® triple quadrupole mass spectrometer (Waters). Prior to infusion, the sample was vortexed, sonicated and filtered using a 0.22 µm Millex-LG filter (Merck-Millipore, Germany). Ionization was achieved using electrospray in positive ion mode. The mass spectrometer was operated in multiple reaction monitoring mode. The entire system was controlled by Masslynx® software (Waters). The molecular weights of the azacitidine and TBDMS being 244.207 g.mol^{-1} and 150.72 g.mol^{-1} respectively, the $[M+H]^+$ at 587.1 m/z and its sodium adduct at 609.03 m/z, confirmed the successful protection of the azacitidine (**Figure S9**).

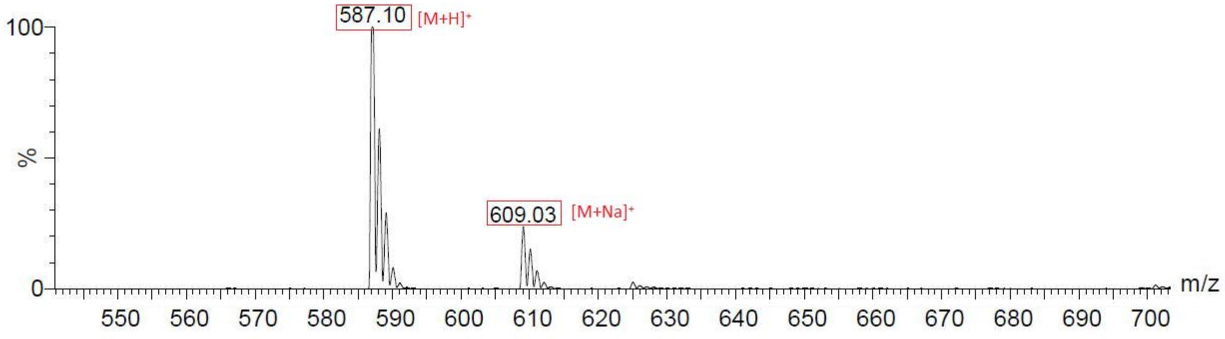

Figure S9: MS spectra of the protected azacitidine.

- **Conjugation of protected azacitidine to the fatty acid**

120 mg of protected azacitidine, 2 equivalents of DHA or EPA, 2.4 equivalents of ET_3N, 2.2 equivalents of EtCOCl in 10 mL of anhydrous THF and DMF were used (in the absence of DMF, the conjugate was not observed, for solubility reasons probably). The fatty acid and ET_3N were mixed together in THF for 15 minutes, followed by cooling down to -10ºC in an acetone ice bath. Then, ethyl chloroformate in THF was added drop-wise and mixed for 15 minutes. Finally, protected azacitidine in DMF was added to the mixture drop-wise and mixed for 10 min, before removing the acetone ice bath and allowing the mixture to reach room temperature and mixed for 72 hours under argon.

The mixture was then dried using a rotary evaporator at 40°C. A 1 mM solution of sodium bicarbonate was added to the mixture and the crude product was extracted using dichloromethane. The organic layer was then washed with brine, dried on magnesium sulfate ($MgSO_4$), then concentrated using a rotary evaporator. The crude product was then purified using a silica gel chromatography eluting with 6 % ethyl acetate in cyclohexane to give the protected azacitidine-DHA/EPA conjugates with a final mean yield of 52 %. Mass spectrometry was used to further confirm the obtained products. The molecular weights of the protected azacitidine and EPA being 586.99 $g.mol^{-1}$ and 302.45 $g.mol^{-1}$ respectively, the $[M+H]^+$ at 871.50 m/z, its sodium adduct at 894.50 m/z, and its potassium adduct at 911.55 m/z confirmed the successful conjugation of the protected azacitidine to the EPA (**Figure S10**) . The molecular weight of DHA being of 328.48 $g.mol^{-1}$, the $[M+H]^+$ at 897.48 m/z, its sodium adduct at 920.40 m/z, and its potassium adduct at 937.49 m/z confirmed achieving the protected azacitidine covalently linked to DHA (**Figure S11**).

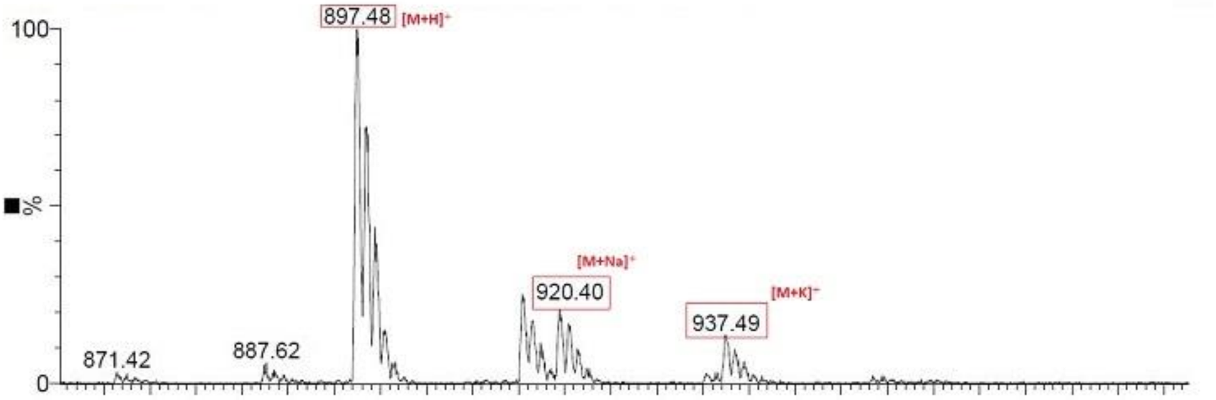

Figure S10: Mass spectra of the protected azacitidine-EPA conjugate.

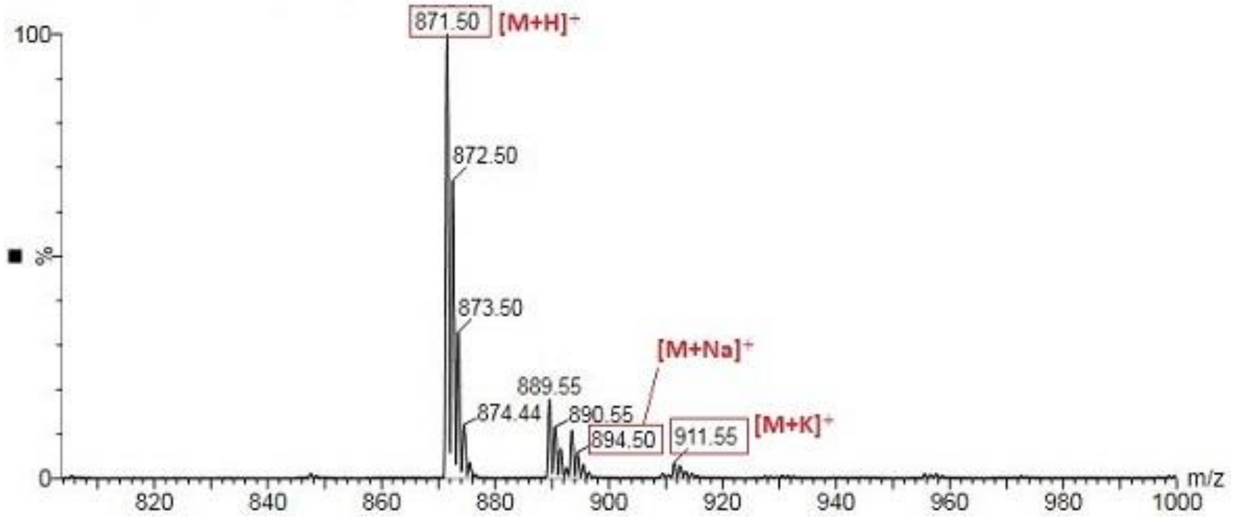

Figure S11: Mass spectra of the protected azacitidine-DHA conjugate.

- **Removal of the silyl protecting group**

After obtaining the protected azacitidine-omega 3 fatty acid conjugates, a final step of deprotection is still needed. To reach this aim, TBAF is known to react with the protected product[1,2], in using 1.1 equivalents per protecting group (3.3 in total). Prior to the reaction, THF and TBAF were mixed together and dried for 72 hours with molecular sieves (3 Å): even if both products were bought in an anhydrous state, several studies have shown that TBAF is an extremely hygroscopic molecule, that will absorb water easily, and even brand new sealed bottles showed a decent amount of water that slowed the reaction down significantly[2].

The protected conjugates were mixed with the dried mixture and allowed to react for 24 hours at room temperature. The mixture was then dried using a rotary evaporator at 40°C. The crude product was analyzed by mass spectrometry and only a partial deprotection was observed. The increase of the reaction time or the amount of added TBAF did not improve the complete deprotection process.

The addition of an equal equivalents of acetic acid to this mixture after doubling the equivalents allowed for the complete deprotection to occur. Precisely, 150 mg of protected azacitidine-fatty acid conjugate was dissolved in 5 mL of THF, followed by the addition of 6 equivalents of TBAF and 6 equivalents of acetic acid, both added drop-wise. The reaction was allowed to stir for 24 hours at room temperature. The mixture was then concentrated using a rotary evaporator at 40°C and the crude product was analyzed using mass spectrometry. The deprotected conjugates were observed for the both EPA and DHA conjugates. The $[M+H]^+$ at 529.48 m/z confirmed obtaining the desired azacitidine-EPA conjugate (**Figure S12**). The $[M+Na]^+$ at 577.13 m/z proved the successful synthesis of the azacitidine-DHA conjugate (**Figure S13**)

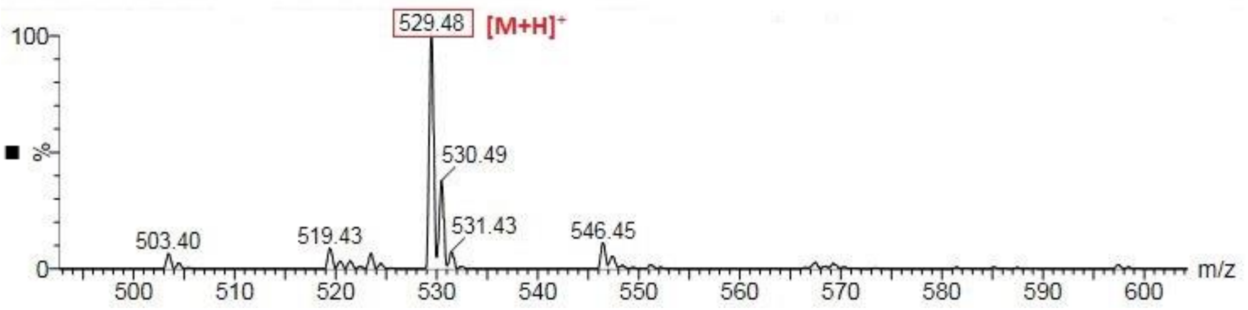

Figure S12: Mass spectra of the azacitidine-EPA conjugate.

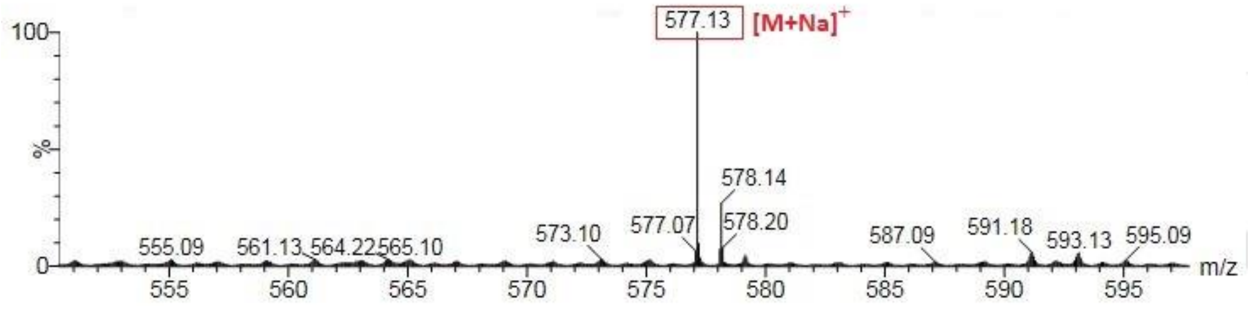

Figure S13: Mass spectra of the azacitidine-DHA conjugate.

After obtaining the crude products of both conjugates, the process of purification was unsuccessful both on silica gel chromatography and reversed phase high-performance liquid chromatography (RP-HPLC), while using a wide range of eluents and conditions. It could be explained by the

extremely close polarity of the final products and the partially deprotected conjugates, which allowed them to elute together.

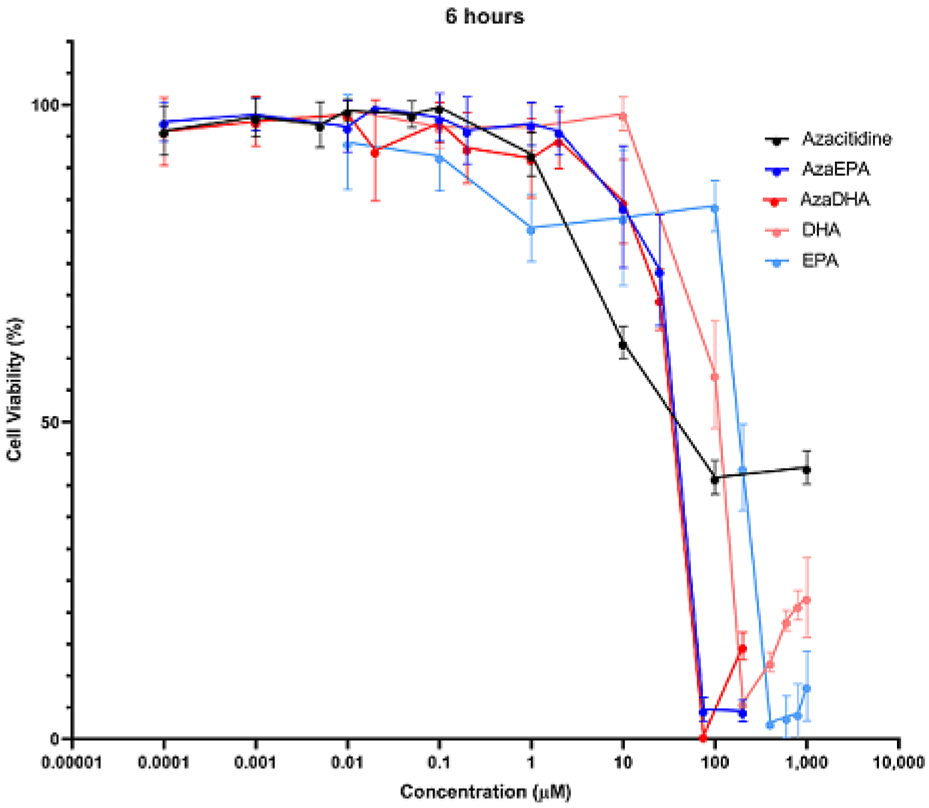

Figure S14: Cytotoxicity studies on the HL-60 cell line of the self-assemblies compared to the free azacitidine and fatty acids at 6 hours, via a MTT assay.

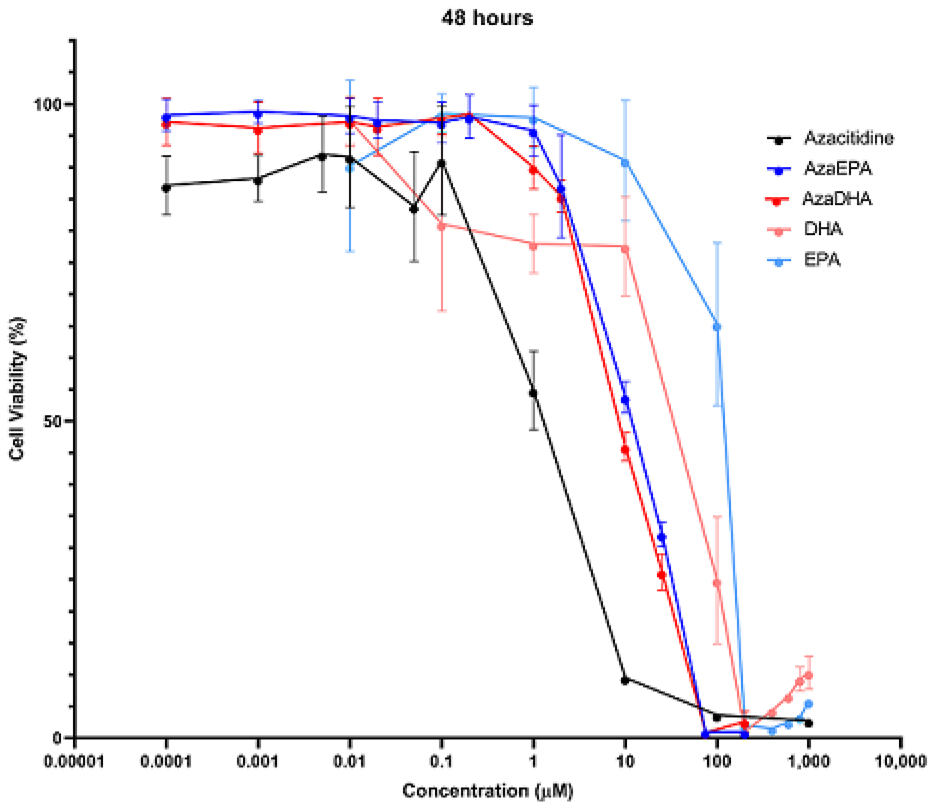

Figure S15: Cytotoxicity studies on the HL-60 cell line of the self-assemblies compared to the free azacitidine and fatty acids at 48 hours, via a MTT assay.

			Size (d.nm):	% Intensity:	St Dev (d.nm):
Z-Average (d.nm):	182,5	Peak 1:	213,8	100,0	90,74
PdI:	0,155	Peak 2:	0,000	0,0	0,000
Intercept:	0,961	Peak 3:	0,000	0,0	0,000
Result quality :	Good				

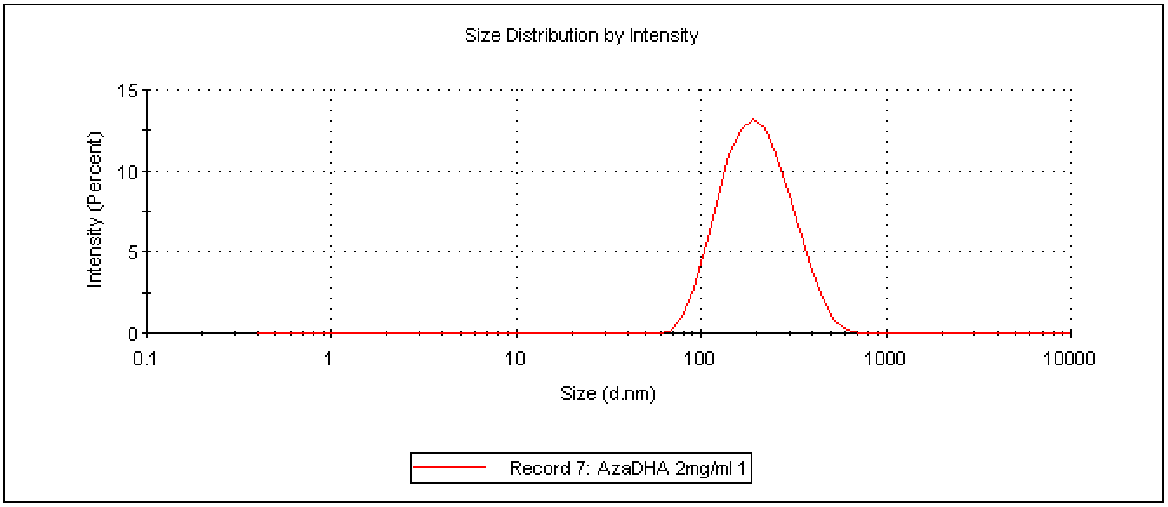

Figure S16: Size distribution by intensity obtained by DLS for AzaDHA self-assemblies on day 7.

			Size (d.nm):	% Intensity:	St Dev (d.nm):
Z-Average (d.nm):	217,0	Peak 1:	258,4	99,3	127,1
PdI:	0,184	Peak 2:	5140	0,7	514,2
Intercept:	0,942	Peak 3:	0,000	0,0	0,000
Result quality :	Good				

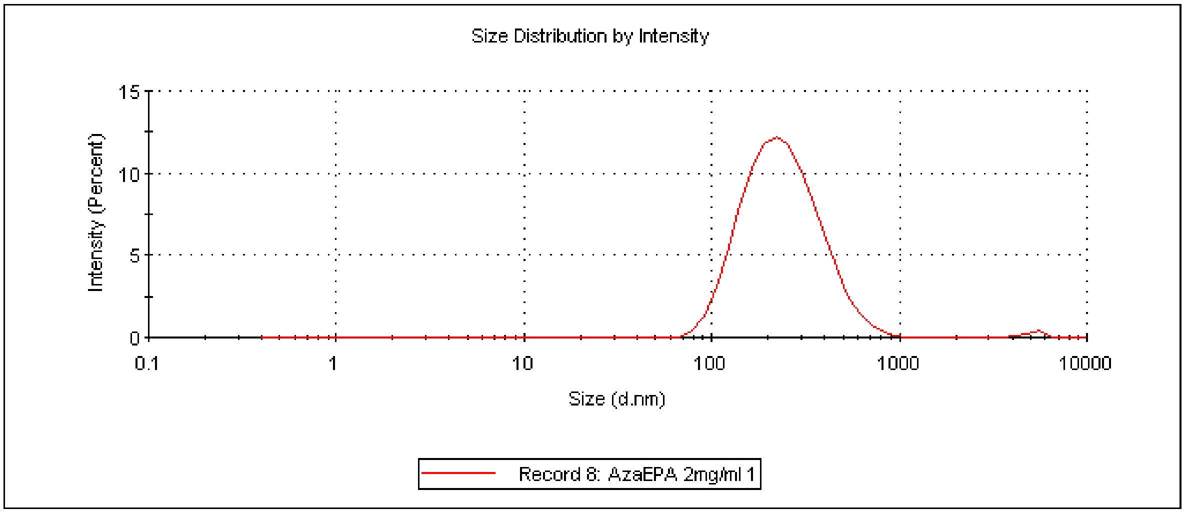

Figure S17: Size distribution by intensity obtained by DLS for AzaEPA self-assemblies on day 7.

References

(1) Gaudin, A.; Yemisci, M.; Eroglu, H.; Lepetre-Mouelhi, S.; Turkoglu, O. F.; Dönmez-Demir, B.; Caban, S.; Sargon, M. F.; Garcia-Argote, S.; Pieters, G.; Loreau, O.; Rousseau, B.; Tagit, O.; Hildebrandt, N.; Le Dantec, Y.; Mougin, J.; Valetti, S.; Chacun, H.; Nicolas, V.; Desmaële, D.; Andrieux, K.; Capan, Y.; Dalkara, T.; Couvreur, P. Squalenoyl Adenosine Nanoparticles Provide Neuroprotection after Stroke and Spinal Cord Injury. *Nature Nanotech* **2014**, *9* (12), 1054–1062. https://doi.org/10.1038/nnano.2014.274.

(2) Higashibayashi, S.; Shinko, K.; Ishizu, T.; Hashimoto, K.; Shirahama, H.; Nakata, M. Selective Deprotection of T-Butyldiphenylsilyl Ethers in the Presence of t-Butyldimethylsilyl Ethers by Tetrabutylammonium Fluoride, Acetic Acid, and Water. *Synlett* **2000**, No. 9, 1306–1308.
